# Ideal cardiovascular health among Ghanaian populations in three European countries and rural and urban Ghana: the RODAM study

**DOI:** 10.1007/s11739-018-1846-6

**Published:** 2018-04-17

**Authors:** Benjamin van Nieuwenhuizen, Mohammad Hadi Zafarmand, Erik Beune, Karlijn Meeks, Ama de-Graft Aikins, Juliet Addo, Ellis Owusu-Dabo, Frank P. Mockenhaupt, Silver Bahendeka, Matthias B. Schulze, Ina Danquah, Joachim Spranger, Kerstin Klipstein-Grobusch, Lambert Tetteh Appiah, Liam Smeeth, Karien Stronks, Charles Agyemang

**Affiliations:** 1 Department of Public Health, Academic Medical Center, Amsterdam Public Health research institute, University of Amsterdam, Meibergdreef 9, 1105 AZ Amsterdam, The Netherlands; 2 Regional Institute for Population Studies, University of Ghana, P. O. Box LG 96, Legon, Ghana; 3 Department of Non-communicable Disease Epidemiology, London School of Hygiene and Tropical Medicine, London, UK; 4 School of Public Health, Kwame Nkrumah University of Science and Technology, Kumasi, Ghana; 5 Institute of Tropical Medicine and International Health, Charité-University Medicine Berlin, Augustenburger Platz 1, 13353 Berlin, Germany; 6 MKPGMS-Uganda Martyrs University, Kampala, Uganda; 7 Department of Molecular Epidemiology, German Institute of Human Nutrition Potsdam-Rehbruecke, Arthur-Scheunert-Allee 114-116, 14558 Nuthetal, Germany; 8 Institute for Social Medicine, Epidemiology and Health Economics, Charité-Universitaetsmedizin Berlin, Luisenstr. 57, 10117 Berlin, Germany; 9 Department of Endocrinology and Metabolism, Charité-Universitaetsmedizin Berlin, Berlin, Germany; 10 Center for Cardiovascular Research (CCR), Charite-Universitaetsmedizin Berlin, Berlin, Germany; 11 Julius Global Health, Julius Center for Health Sciences and Primary Care, University Medical Center Utrecht, Utrecht University, Utrecht, The Netherlands; 12 Division of Epidemiology and Biostatistics, School of Public Health, Faculty of Health Sciences, University of the Witwatersrand, Johannesburg, South Africa; 13 Department of Medicine, Komfo Anokye Teaching Hospital, Kumasi, Ghana

**Keywords:** Ideal cardiovascular health, Cardiovascular disease, Ethnic minority groups, Migration, Ghana, Sub-Saharan Africa, Europe, RODAM study

## Abstract

Cardiovascular health (CVH) is a construct defined by the American Heart Association (AHA) as part of its 2020 Impact Goal definition. CVH has, until now, not been evaluated in Sub-Saharan African populations. The aim of this study was to investigate differences in the prevalence of ideal CVH and its constituent metrics among Ghanaians living in rural and urban Ghana and Ghanaian migrants living in three European countries. The AHA definition of CVH is based on 7 metrics: smoking, body mass index, diet, physical activity, blood pressure, total cholesterol, and fasting plasma glucose. These were evaluated among 3510 Ghanaian adults (aged 25–70 years) residing in rural and urban Ghana and three European cities (Amsterdam, London and Berlin) in the multi-centre RODAM study. Differences between groups were assessed using logistic regression with adjustments for gender, age, and education. Only 0.3% of all participants met all 7 metrics of the AHA’s definition of ideal CVH. Compared to rural Ghana (25.7%), the proportions and adjusted odds ratio (OR) of individuals who had 6–7 CVH metrics in the ideal category were substantially lower in urban Ghana, (7.5%; OR 0.204, 95% CI 0.15–0.29), Amsterdam (4.4%; 0.13, 0.08–0.19), Berlin (2.7%; 0.06, 0.03–0.11), and London (1.7%; 0.04, 0.02–0.09), respectively. The proportion of ideal CVH for the various metrics ranged from 96% for all sites in the smoking metric to below 6% in the diet metric. The proportion of ideal CVH is extremely low in Ghanaians, especially among those living in urban Ghana and Ghanaian migrants in Europe.

**Electronic supplementary material:**

The online version of this article (10.1007/s11739-018-1846-6) contains supplementary material, which is available to authorized users.

## Introduction

Cardiovascular diseases (CVD), especially ischaemic heart disease and stroke, are the leading cause of morbidity and mortality worldwide [1]. Although CVD mortality rates have decreased in the USA [2] and in Europe [3], the rise of obesity and type 2 diabetes threaten to reverse this trend. Ethnic minority populations in Europe appear to exhibit higher rates of CVD and its associated risk factors such as type 2 diabetes and obesity, than the European host populations [4]. Furthermore, low- and middle-income countries (LMICs) are experiencing a steep surge in CVD prevalence, while already contributing 80% to the global burden of CVD [5]. This high CVD burden in LMICs is associated with changes in living and work environments following urbanisation, increasingly sedentary lifestyle, westernised diet as well as increased rates in diabetes, obesity, and smoking [6].

As part of its impact goals, the American Heart Association (AHA) in 2010 included the goal to increase the overall cardiovascular health (CVH) within the whole (US) population [7]. To realise this goal, the AHA created a quantifiable construct, which included four behavioural (smoking, body mass index [BMI], physical activity [PA], and diet) and three biomedical (blood pressure, total cholesterol, and fasting plasma glucose [FPG]) risk factors for CVD. Thresholds were applied to define three categories (poor, intermediate, and ideal) for each constituent ‘metric’ and for overall CVH (Supplementary Table 1). Several studies have been carried out to show the distribution of the ideal CVH metrics, comparing the CVH in different ethnic minority populations in the USA [8, 9]. Folsom et al. [8] show that African Americans are less likely to have overall ideal CVH than White Americans, and Bambs et al. [9] show that White Americans are more likely to fall within the ideal category for each individual CVH metric than African Americans except for total cholesterol.

**Table 1:** Baseline characteristics of study population by gender and by site

Baseline characteristics	All sites	Amsterdam	Berlin	London	Ghana Urban	Ghana Rural
Women	*n* = 2166	*n* = 532	*n* = 211	*n* = 501	*n* = 596	*n* = 326
Mean age (SD)	45 (11)	44 (9)	42 (11)	47 (11)	44 (11)	45 (13)
Gender (%)	2166 (62)	532 (60)	211 (46)	501 (63)	596 (72)	326 (60)
Current smokers (%)	14 (1)	6 (1)	6 (1)	1 (0)	1 (0)	0
Mean height (cm) (SD)	160 (6)	161 (6)	163 (6)	161 (6)	159 (6)	158 (7)
Mean weight (kg)	73 (16)	78 (15)	75 (12)	80 (15)	71 (15)	59 (13)
Mean BMI (SD)	29 (6)	30 (5)	28 (5)	31 (5)	28 (5)	24 (5)
Education above college	194 (9)	20 (4)	21 (10)	127 (26)	18 (3)	8 (2)
Education below college	1955 (91)	505 (96)	189 (90)	367 (74)	578 (97)	316 (98)
Alcohol (g/day) (SD)	2 (9)	3 (9)	6 (18)	2 (8)	1 (3)	1 (4)
Men	*n* = 1344	*n* = 348	*n* = 250	*n* = 298	*n* = 234	*n* = 214
Mean age (SD)	47 (12)	48 (9)	45 (12)	46 (12)	46 (12)	48 (14)
Gender (%)	1344 (38)	348 (40)	250 (54)	298 (37)	234 (28)	214 (40)
Current smokers (%)	86 (6)	24 (3)	34 (7)	4 (1)	9 (1)	15 (3)
Mean height (cm) (SD)	171 (7)	171 (6)	173 (6)	171 (6)	169 (7)	168 (7)
Mean weight (kg)	75 (15)	79 (13)	79 (13)	80 (12)	69 (13)	60 (11)
Mean BMI (SD)	25 (4)	27 (4)	26 (4)	27 (4)	24 (4)	21 (3)
Education above college	264 (20)	31 (9)	46 (18)	143 (49)	27 (12)	17 (8)
Education below college	1073 (80)	315 (91)	204 (82)	150 (51)	207 (88)	197 (92)
Alcohol (g/day) (SD)	10 (27)	9 (16)	22 (41)	3 (9)	3 (11)	10 (36)

All variables reported as a means and standard deviation (SD) in brackets or frequencies and percentage (%) in brackets

Currently, data on CVH among sub-Saharan African populations in the African region are scarce [10], and lacking for sub-Saharan African populations living in Europe. Although African Americans may share common ancestry with sub-Saharan African migrants in Europe, these populations differ considerably in socio-cultural aspects such as culture, language, diet, and socio-economic status as well as environmental factors including their migration history and the geographical location in which they reside [11, 12]. In addition, African Americans differ genetically from sub-Saharan African migrants in Europe due to genetic admixture [13]. Most of these factors have an influence on CVD [10] and thus probably also CVH. Therefore, sub-Saharan African migrants in Europe are likely to present different prevalence rates of ideal CVH than African Americans [14]. Furthermore, evidence suggests important differences in CVD outcomes among sub-Saharan African populations residing in different European countries [10]. It is unknown whether the prevalence of ideal CVH also varies among a particular migrant population living in different European countries. It is also unknown whether the prevalence of ideal CVH varies between migrants residing in European countries and their counterparts that remained in their country of origin.

Our study aim was to assess differences in the prevalence of CVH, as defined by the AHA, across a relatively homogenous population living in five different contexts (urban Ghana, rural Ghana, Amsterdam, Berlin, and London).

## Methods

### Study population and study design

The data used for this study come from the Research on Obesity and Diabetes among African Migrants (RODAM) study [15]. Detailed description of the study, including the rationale, conceptual framework, design, and methodology of the RODAM study, has been given elsewhere [15–17]. In short, the study was carried out between 2012 and 2015, and it includes a relatively homogenous population of Ghanaian individuals residing in five study sites: rural Ghana, urban Ghana, Amsterdam, Berlin, and London. Urban Ghanaian subjects were recruited from two cities within the Ashanti region: Kumasi and Obuasi and rural Ghanaians were recruited from 15 villages within the Ashanti region. Ghanaian migrants residing in the European sites were selected from a compiled list of individuals gleaned from population registries or Ghanaian community organizations. Ethical approval was obtained from the respective ethics committees at all sites before data collection began in each country. Informed written consent was also obtained from each participant prior to enrolment in the study. The response rates in Ghana were 76% in the rural areas and 74% in the urban areas. The response rates in London and Berlin were 75% and 68%, respectively, among individuals who were registered in the various Ghanaian organizations and were invited to participate in the study. In Amsterdam, 67% of the contacted Ghanaians replied to the invitation and 53% of these subsequently took part in the study. The total number of cases within the RODAM study is 5898 [16]. For this current analysis, 174 individuals were excluded for falling outside the age range of 18–70. Of the remaining 5724 participants, 1966 were excluded due to having pre-existing CVD, as assessed by the Rose Angina questionnaire and a cerebrovascular accident questionnaire. Subsequently, 248 participants were excluded from the remaining 3758 for having systematically missing data for both the PA and diet questionnaires. This resulted in the inclusion of 3510 individuals in this study.

### Measurements

Physical examinations and questionnaires were completed according to standardized operational procedures across all study sites. Data on socio-demographics, health behaviour, dietary intake, and medical history were obtained by questionnaire. The health behaviour variables included smoking status, length of time since cessation of smoking and total physical activity (PA) per week. The WHO STEPS questionnaire was used to derive PA in metabolic equivalent of task (MET) h/week, including PA at work, while commuting and in leisure time [18]. Smoking status was assessed as a positive reply to the question ‘Do you smoke at all?’ Food intake was assessed using a standardized Food Propensity Questionnaire (Ghana-FPQ) based on the multi-language, semi-quantitative European Food Propensity Questionnaire (EFPQ) [19]. The Ghana-FPQ covers 134 food items in the preceding 12 months. The German Nutrient Database (BLS3.01) (2010) and the West African Food Composition Table (2012) were used to translate usual food intake (g/day) into energy consumption and intake of nutrients. The diet variables included the consumed amounts and frequency of the following: whole-grain bread and cereals (g/day), fruit (g/day), vegetables (g/day), fish (g/day), condiments (g/day), sugar-sweetened beverages (g/day), alcohol (g/day), and total energy intake (kcal/day). Sodium intake was estimated from the food frequency questionnaire (FFQ) in combination with food composition databases, which provided estimates of sodium content in each food assessed on the questionnaire. Physical measurements included anthropometrics and blood pressure. All the anthropometrics were measured two times by the same assessor and the mean of the two measurements was used for analyses. Weight was measured using SECA 877 scales to the nearest 0.1 kg in light clothing and without shoes. Height was measured with a portable stadiometer (SECA 217) to the nearest 0.1 cm without shoes. Body mass index (BMI) was calculated as weight (kg) divided by height squared (m^2^). Blood pressure was measured three times with a validated semi-automated device (The Microlife WatchBP home) with appropriate cuffs in a sitting position after at least 5 min rest. The mean of the last two readings was used in the analyses.

Fasting venous blood samples were collected by trained research assistants in all sites. All the samples were processed and divided into aliquots immediately after collection, and then, temporarily stored at the local research location at − 20 °C according to the standard operational procedures. The samples were subsequently transported to the local research centres´ laboratories, where they were checked, registered, and stored at − 80 °C. The stored blood samples from the local research centres were finally transported to Berlin, Germany for biochemical analyses to avoid intra-laboratory variability. The concentration of fasting plasma glucose was assessed using an enzymatic method (hexokinase) and the total cholesterol concentration was determined using colorimetric test kits. All biochemical analyses were done using an ABX Pentra 400 chemistry analyzer (HORIBA ABX SAS, Montpellier, France).

### Cardiovascular health definitions

Supplementary Table 1 shows the AHA definitions [7], which were followed by this study, for the three categories (ideal, intermediate, and poor) for each of the 7 CVH metrics and overall CVH. The five dietary components were defined by an individual consuming the following:

- ≥ 450 g of fruits and vegetables per day;
- ≥ two 100 g portions of fish a week;
- ≥ three 30 g equivalent servings of fibre-rich whole grains per day;
- ≤ 1500 mg sodium per day;
- ≤ 450 kcal sugar-sweetened beverages: (1 L) per week.

The definitions of the biomedical metrics did not “include those, who achieve ideal levels of cardiovascular health factors through medication use (i.e., lipid-lowering, antihypertensive, or hypoglycemic agents) [7].” The thresholds defining the poor and intermediate categories remain the same for those on such medication.

For this study, the data collected by RODAM were converted into units that satisfied the AHA definition for CVH as closely as possible [7]. The FPG, total cholesterol and blood pressure, BMI, and smoking data were provided in units that the AHA thresholds could be directly applied to. The threshold weights of all the diet metric components apart from fruit and vegetables were recalculated to produce a daily threshold of g/day, as was recorded in the RODAM database. As there was no standardized weight for a cup of fruits and vegetables, which forms part of the AHA definition of daily fruit and vegetable intake, this study used the Dutch nationally recommended fruit and vegetable intakes to define the threshold in grams per day (200 g of fruit and 250 g of vegetables [20]). A total number of minutes of PA per week were estimated from moderate and vigorous activity both in recreational and work settings, in which the vigorous activity was weighted twice as strongly as the moderate activity. To create three categories (poor, intermediate, and ideal) for overall CVH, the AHA definition was followed (Supplementary Table 1).

### Statistical analysis

To avoid the bias associated with the remaining missing data (after the exclusion of cases systematically missing data in all constituent variables for PA and diet, as explained under ‘study sample and study design’), multiple imputations were used for variables with missing values. Missing data were primarily due to non-response on the diet or PA questionnaires. The proportion of missing values among 3510 included individuals was 17.6% in the diet metric, 4.3% in the PA metric and 3% in the FPG metric, while less than 1% of the remaining metrics had missing values. Multiple imputations were carried out on the summary variables, e.g., the CVH metrics expressed in three tiers (poor, intermediate, and ideal) on the basis of the correlation of missing variables with other participant characteristics. Although usually, 5–10 data sets are deemed sufficient for multiple imputation, generating 20 data sets has been suggested, recently, to reduce sampling variability from the imputation process [21]. In addition, we added variables related to covariates as predictors to the imputation model to increase the plausibility of the missing-at-random assumption. The method used was the Markov chain Monte Carlo (MCMC) algorithm, embedded in SPSS (IBM analytics). The difference in means between the original data and the pooled imputation data sets was less than 0.006 for every metric. This study reports the pooled results of analyses performed in each of the 20 imputed data sets.

Baseline characteristics of the study population were calculated as means or numbers with corresponding standard deviations or percentages, respectively. A Kruskal—Wallis test was carried out to ascertain whether the proportions of the three categories differed significantly across the study sites. Subsequently, the distribution of the proportion of individuals with 0–7 CVH metrics in the ideal category in each site was dichotomised to those with 0–5 and those with 6–7. Having six or seven ICVH metrics was chosen to define ‘ideal’ CVH because of the small number of participants having all seven ideal metrics (0.3%), as has been opted for in the previous studies [9]. Binomial logistic regression was carried out to compare the odds of individuals in each site of having 6–7 ideal CVH metrics compared to the reference population of rural Ghana, taking into account gender, age, and education level as confounding factors. Rural Ghana was chosen as the reference, with which the other sites were compared, due to rural Ghana being conceptually the environment of migratory origin, while the urban environments were conceptually the migratory destination. All statistical procedures were carried out in the program SPSS version 22.0 (IBM Corp., Armonk, NY, USA).

## Results

### Baseline characteristics of study population

Table 1 summarises the baseline demographic variables for the study population on which analyses were carried out. 3% of the overall study population were smokers which ranged from 1% in urban Ghana and London to 9% in Berlin. Mean BMI was 27 (± 5) kg/m^2^, ranging from 23 kg/m^2^ in rural Ghana to 30 kg/m^2^ in London. Women had a higher BMI than men in all sites; the difference in BMI ranged from two points in Berlin to four points in London. Of all the participants, 9% had completed education above college or a high-level vocational education; this ranged from 5% in rural and urban Ghana to 34% in London. Men attained this level of education more often than women.

#### CVH metrics

The distribution of the CVH categories (poor, intermediate, and ideal) is shown for each of the 7 CVH metrics (Supplementary Figs. 1–7) and overall CVH (Table 2), comparing across the five study sites as well as the average across all sites. For figures, results have been reported separately by gender, with panel A showing the results for women and panel B for men.

**Table 2:** Proportion of individuals with 0–7 CVH metrics in the ‘ideal’ category per site and for all sites in the RODAM study

	All sites		Amsterdam		Berlin		London		Ghana urban		Ghana rural	
ICVH metrics	%	95% CI	%	95% CI	%	95% CI	%	95% CI	%	95% CI	%	95% CI
Women												
0	0.0	0.0–0.2	0.0	0.0–0.6	0.0		0.0		0.1	0.0–0.5	0.0	
1	3.2	3.2–5.0	3.9	3.9–7.9	5.2	2.5–8.7	4.5	4.2–8.5	2.0	0.8–3.2	0.9	0.0–2.1
2	14.3	14.7–17.7	15.4	15.6–22.5	18.3	13.0–23.7	18.5	18.8–25.9	14.9	12.1–17.7	2.7	1.5–5.5
3	28.7	26.7–30.6	30.8	26.4–34.4	30.6	23.9–36.9	37.1	31.7–40.0	27.7	24.3–31.4	13.1	10.5–17.9
4	28.6	25.9–29.7	27.2	21.7–29.0	27.9	21.4–34.0	29.8	24.3–32.1	30.8	27.0–34.4	25.2	20.8–30.6
5	17.7	15.0–18.1	17.0	11.6–17.5	14.6	10.2–20.1	8.6	4.5–9.0	18.2	15.2–21.1	33.8	28.3–38.3
6	7.2	5.5–7.6	5.7	2.8–6.3	2.0	0.5–4.0	1.5	0.0–1.6	6.0	4.2–8.0	23.9	18.0–26.8
7	0.3	0.1–0.5	0.0		1.4	0.0–3.2	0.0		0.3	0.0–0.8	0.4	0.0–1.0
Men												
0	0.1	0.0–0.4	0.6	0.0–1.5	0.0		0.0		0.0		0.0	
1	4.7	4.4–6.9	7.2	6.2–12.7	6.4	3.6–9.3	5.9	4.7–10.9	1.8	0.4–4.1	0.0	
2	15.1	14.4–18.5	20.5	0.18–27.5	23.7	18.9–29.5	17.2	15.1–24.1	7.9	4.5–11.1	1.4	0.0–3.3
3	25.5	23.7–28.6	28.2	0.25–35.6	29.5	24.0–35.6	30.7	24.7–35.4	26.2	20.8–31.7	8.6	5.6–13.5
4	27.6	23.7–28.5	28.0	0.19–28.4	24.4	18.9–29.3	29.6	22.9–33.2	30.3	24.8–36.2	25.2	19.2–31.0
5	19.2	16.2–20.3	13.2	0.8–14.5	13.9	9.8–18.7	14.6	9.4–16.9	23.5	17.9–29.1	36.8	30.2–43.1
6	7.5	5.7–8.6	2.0	0.3–2.4	2.1	0.4–3.8	2.0	0.3–3.4	10.3	6.5–14.5	27.0	21.4–32.8
7	0.3	0.0–0.5	0.3	0.0–0.9	0.0		0.0		0.0		1.0	0.0–2.4

All values in this table are percentages and corresponding 95% confidence intervals (CI) of each of the CVH metrics in the ideal category (0–7)

### Smoking

The vast majority of all participants were in the ideal category for the smoking metric (99% in women and 92% in men). Berlin had the highest proportion of current smokers (3% in women and 14% in men) and men (6%) smoked more than women (1%) across all sites (Supplementary Fig. 1).

### BMI

More men (47%) were in the ideal category for BMI than women (27%). In both men and women, there was a gradual decrease in the proportion of individuals within the ideal category from rural Ghana to urban Ghana to any of the European sites. London had the lowest proportion of individuals in the ideal category for BMI in both men and women (Supplementary Fig. 2).

### Physical activity

The PA metric was scored more frequently in the ideal category than in the poor category except for women living in Berlin, where these two categories were scored equally. More men (70%) were in the ideal category for PA than women (64%) across all sites (Supplementary Fig. 3). A similar gradient in the ideal category was present in PA from rural Ghana to urban Ghana and Europe except for women in Amsterdam, who had a higher proportion in the ideal category (72%) compared to women in urban Ghana (60%).

### Diet

The proportion of individuals in the poor category for the diet metric across all sites was 12% for women and 21% for men. Berlin had the highest proportion of individuals in the poor category, with 20% of women and 36% of men. The proportions of individuals in the poor category for the diet metric were relatively similar between urban (4% for women and 11% for men) and rural Ghana (8% for women and 13% for men), while this is substantially higher in Berlin and London (19% for women and 29% for men) (Supplementary Fig. 4). The proportion of individuals in the ideal category is generally low, ranging from 0% in men from urban Ghana to 13.5% in women living in Amsterdam.

### Total cholesterol

The majority of individuals across all sites were in the ideal category for the cholesterol metric (55% for women and 58% for men). In rural Ghana, the proportion of individuals in the ideal category was substantially higher than the other sites, and shows the largest gender difference (67% in women and 82% in men). The other sites had relatively similar proportions of individuals within CVH categories for total cholesterol, although women in Amsterdam had a higher proportion within the ideal category (60%) compared to Berlin (51%), London (51%) and urban Ghana (50%) (Supplementary Fig. 5).

### Blood pressure

The minority of women (33%) and men (20%) were in the ideal category for the blood pressure metric (20%). A similar gradient in the ideal category was present for the blood pressure metric as is the case in the BMI metric (rural Ghana > urban Ghana > European sites). Berlin had the lowest proportion of women (19%) in the ideal category, and London had the lowest proportion of men (9%) in the ideal category compared to the other sites (Supplementary Fig. 6).

### Fasting plasma glucose

The majority of women (81%) and men (72%) were in the ideal category for the FPG metric. Amsterdam had the lowest proportion of women (71%) and men (58%) in the ideal category of FPG among the sites, while the other sites had relatively similar proportions in each of the CVH categories, except women living in Berlin, of whom 90% were in the ideal category (Supplementary Fig. 7).

### Overall CVH

**Fig. 1:**
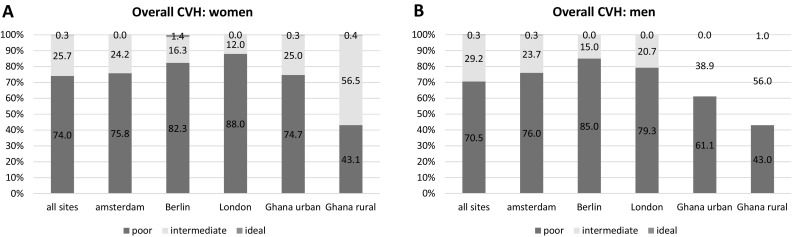
Distribution of overall CVH (ideal, intermediate and poor) in women (**a**) and men (**b**). Each bar represents one of the five study sites except the first bar, which is an average of all sites

Less than 1% of all participants were in the ideal category for overall CVH (Fig. 1). A gradient in the poor category is present for overall CVH from rural Ghana through urban Ghana to Europe. In the European sites, over three quarters of both men and women were in the poor category. The population with the highest proportion in the poor category was women in London (88%).

The proportion (and its confidence interval) of the number of CVH metrics for each site and for the average across all sites, which are in the ideal category, is displayed in Table 2. Rural Ghana had the highest proportion (25.7%), followed by urban Ghana (7.5%), and the European sites had the lowest proportion of individuals with 6–7 ICVH metrics (4.4% in Amsterdam, 2.7% in Berlin, and 1.7% in London). In rural and urban Ghana, the majority of individuals had four or more ICVH metrics, while in Amsterdam, Berlin, and London, the majority of individuals had three or less ICVH metrics.

Results of the Kruskal–Wallis test show that the proportions of the poor, intermediate, and ideal CVH categories were significantly different across the five study sites for all of the CVH metrics and overall CVH (*p* < 0.001). The results of the regression analysis showed that in comparison with rural Ghanaians and after adjustment for age, gender and education level, urban Ghanaians had 80% lower odds of having 6 or more components of ideal cardiovascular health (OR 0.20, 95% CI 0.15–0.29; *p* < 0.001) (Table 3). Compared to rural Ghanaians, Ghanaian migrants residing in Amsterdam, Berlin, and London had lower odds of having ≥ 6 components of ideal CVH after adjustment for age, gender, and education level. Migrants in Amsterdam had 87%, (OR 0.13, 95% CI 0.08–0.19; *p* < 0.001) lower odds, migrants in Berlin had 94%, (OR 0.06, 95% CI 0.03–0.11; *p* < 0.001) lower odds, and migrants in London had 96%, (OR 0.04, 95% CI 0.02–0.09; *p* < 0.001) lower odds of having ≥ 6 components of ideal CVH independent of age, gender, and education level.

**Table 3:** Crude and adjusted odds ratios of ideal cardiovascular health by locality—RODAM study

	Crude		Model 1		Model 2	
	OR (95% CI)	*p*	OR (95% CI)	*p*	OR (95% CI)	*p*
Rural Ghana	Reference		Reference		Reference	
Urban Ghana	0.233 (0.169–0.322)	< 0.001	0.203 (0.145–0.284)	< 0.001	0.204 (0.145–0.286)	< 0.001
Amsterdam	0.132 (0.088–0.197)	< 0.001	0.126 (0.083–0.190)	< 0.001	0.125 (0.082–0.190)	< 0.001
Berlin	0.081 (0.044–0.148)	< 0.001	0.059 (0.032–0.110)	< 0.001	0.059 (0.031–0.111)	< 0.001
London	0.050 (0.026–0.095)	< 0.001	0.047 (0.025–0.090)	< 0.001	0.043 (0.021–0.087)	< 0.001

Model 1 was adjusted for gender and age; Model 2 was adjusted for gender, age and education level

*OR* odds ratio, *CI* confidence interval

## Discussion

### Key findings

A very low prevalence of ideal overall CVH was found among Ghanaians resident in multiple sites in Europe and Ghana even after adopting a more lenient definition. Ideal CVH decreased with a gradient from rural Ghana to urban Ghana to the European cities. Out of the European cities, Amsterdam had the highest proportion of individuals with ideal CVH, which was followed by Berlin, while London had the lowest proportion of individuals with ideal CVH. Out of the European cities, Ghanaians living in Amsterdam had the highest proportion of individuals in the ideal category for PA and diet, but had a slightly lower proportion of individuals in the ideal category for FPG compared to other sites. Berlin Ghanaians had the highest proportion of individuals in the ideal category for BMI, but had the highest proportion of current smokers. The blood pressure metric was relatively similar across the European sites, and the total cholesterol metrics did not vary substantially across all the urban sites (including urban Ghana). Moreover, a substantially higher proportion of individuals were in the poor category for the diet metric in Berlin and in London compared to the remaining sites.

### Discussion of the key findings

The results of the ideal CVH gradient from the rural Ghana through urban Ghana to Europe are consistent with the studies on rural through urban and high-income countries on cardiovascular disease risk factors [22–26]. This is very much a tribute to the impact of environmental factors on the constituent CVH metrics. An important underlying factor for this trend is the change in health-related behaviour that commonly accompanies migration, such as increased sedentary lifestyles, a decrease in PA, and a less healthy diet [25, 27–30].

The results for the individual metrics are in accordance with the previous findings in the RODAM population [31–33]. The high level of physical activity in Amsterdam may be associated with the high proportion of physically demanding jobs reported by the Amsterdam Ghanaian community possibly due to low level of education among this group [34]. In London and Berlin, on the other hand, the Ghanaian community has a higher level of education, and thus may have had jobs that were less physically demanding, compared with those living in Amsterdam. The differences in proportions of individuals within the ideal category of the BMI and smoking metrics, amongst the European sites, may be explained by the differences in contextual factors influencing BMI and smoking in the countries of settlement. The Netherlands has a mean BMI of 24.1, Germany has a mean BMI of 25.3, and the UK has a mean BMI of 26.2 [35]. Smoking rate is highest in Germany (32.4%), followed by The Netherlands (26.2%) and UK (19.9%) according to the WHO Global Health Observatory (GHO) 2015 age-standardized data (http://www.who.int/gho/tobacco/use/en/). The lower proportion of individuals within the ideal category for FPG that we observed in Amsterdam compared to the other European sites does not reflect prevalence rates in the general population nor the constituent diet metric [36]. Less healthy levels of FPG in the Ghanaian population in Amsterdam may be due to dietary habits not captured by the AHA definition of the diet metric that was used for this study.

Several studies have assessed CVH within populations in Europe. However, due to some not reporting overall CVH or using a different definition for overall CVH [37–43], it has not been possible to compare our results with every population [41–43] in which CVH prevalence has been reported in Europe. Graciani et al. [37] reported CVH prevalence in a nationally representative Spanish population in which 3.6% of the population were found to have 6 or 7 metrics in the ideal category. This is similar to our results for the mean across the European sites in RODAM, where 3% of the population had 6 or 7 metrics in the ideal category. Wilsgaard and colleagues [38] found a lower proportion (2.4%) of individuals with 6 or 7 metrics in the ideal category among residents of Tromsø, Norway aged 30–79 years. Crichton et al. [39] conducted a study on a nationally representative sample in Luxembourg, where 20.6% of the study population were found to have 6 or more metrics in the ideal category. However, this study utilised two different diet measures, a recommended food score and a non-recommended food score, resulting in there being 8 CVH metrics rather than 7. As both diet metrics were scored in the ideal category frequently (≈ 40%), and as they were likely to be scored similarly by individuals, the addition of the extra diet metric is likely to have made the inclusion into the ideal category of overall CVH more lenient. Moreover, participants of the Paris Prospective Study III aged 50–75 years, have about 0.2% overall ideal CVH when having 5–7 ideal metrics categorised as ideal CVH [40]. Another study from Bosnia and Herzegovina shows a higher proportion (about 7% in women and 3% in men) of individuals with 6 or 7 metrics in the ideal category, which may be attributed to the younger participants (18–39 years compared to above 40 years) [41]. O’flynn et al. [42], in a population of middle-aged Irish adults, only included individuals with 7 ideal CVH metrics in the ideal category of overall CVH, and found that no-one in this study population met those requirements. In the European sites of RODAM, on the other hand, 0.2% of the population had seven metrics in the ideal category.

A recent study in the UK reported a lower prevalence of overall ideal CVH than we found in RODAM [43]. The proportion of ideal CVH in the EPIC-Norfolk cohort was 2.8%, while in the London site of the RODAM study, 5.9% were in the ideal category, after applying the same threshold for the ideal category to our data. In the EPIC-Norfolk study, the definition of the ideal category of overall CVH is 12–14 points on a 0–14-point score, where the score is the sum of the individual metrics (poor = 0, intermediate = 1 and ideal = 2). The prevalence of obesity in EPIC-Norfolk was 14%, which is substantially lower than the 42% found in this study, among Ghanaians living in London. On the other hand, for the cholesterol metric, 53% of Ghanaians living in London were observed to be in the ideal category in this study, while only 20% of the EPIC-Norfolk cohort were in the ideal category. Smoking is also far less prevalent in the London site of the RODAM cohort than in the EPIC-Norfolk study cohort. Blood pressure, diet, physical activity, and FPG levels are similar both in proportions of individuals in the ideal and poor categories between the London site of the RODAM study and the EPIC-Norfolk cohort. These findings are somewhat surprising considering that previous reports found a higher prevalence of hypertension and type 2 diabetes among African populations compared to the European host populations in Europe [44–46]. The lack of expected disparity between these cohorts in the FPG and blood pressure metrics might be attributable to the high level of education in the London site of RODAM, which may also have had an influence on health behaviours of the Ghanaian population. It may also be due to other contextual factors affecting health outcomes in the UK [47].

The African migrant population in Europe seems to have better CVH compared to African Americans in the US. Comparing the number of CVH metrics in the ideal category between the European sites in the RODAM study to the African Americans in the ARIC cohort shows a higher proportion of individuals in the RODAM cohort (44%) with 4–7 ideal CVH metrics compared to the ARIC cohort (16%) [8]. Comparing the average of the European sites in the RODAM cohort to the African American population assessed by Bambs et al. [9] shows that the RODAM population has higher proportions of individuals in the ideal category across all CVH metrics except diet. Furthermore, RODAM has lower proportions of individuals in the poor category in all metrics except PA than the African American population. The lower proportions of individuals in the ideal category in studies by Folsom et al. and Bambs et al. compared to the RODAM cohort may be partially accounted for by the generally lower ideal CVH prevalence seen in the US compared to Europe. This is likely caused, in part, by the relatively low accessibility of healthy foods and high accessibility of unhealthy foods [48, 49]. In a literature review conducted by our own group [van Nieuwenhuizen et al. unpublished (2017)], there was a higher proportion of individuals in Europe with 4–7 CVH metrics in the ideal category (44.6%) than in North America (36.7%). On the other hand, there may be environmental factors associated specifically with the African American population in the US that has an influence on this disparity as well. For example, there is clearly a historical difference in the migratory origin between African Americans and African Europeans, which is likely to have left socio-cultural influences to this day. Furthermore, African Americans have been in the US for several generations, while Ghanaians in the RODAM study are mostly first generation migrants. This has an impact on the effect of acculturation of the migrant population and the ‘host’ population simply due to the amount of time that contact has taken place. The observed important differences in CVH among these African populations further demonstrate the need to pay attention to the diversity within the African descent populations in health studies [50].

Although overall CVH appears not to differ between men and women, the pattern of metrics which add up to the total score does differ across the sexes. Gender differences found in the individual metrics across the study sites is in accordance with gender differences found in the constituent risk factors. Women have been reported to adhere more prevalently to healthy diets than men [51]. Obesity is more common in women than in men globally [52]. Engaging in a high amount of physical activity is more prevalent in men than in women. Hypertension, impaired fasting glucose, and smoking are generally higher in men than in women worldwide [53–55]. However, smoking prevalence in many high-income countries is equal in men and women, which may suggest that as migrant groups in high-income countries culturally converge more over time towards the host population, gender differences may decrease [56].

### Strengths and limitations

The main strength of the RODAM study is the use of well-standardized approaches across the various study sites. Another unique strength of this study is the homogeneity of the study population across the different settings in Africa and Europe. Thus, this study was able to examine the difference of CVH prevalence across different living environments without a high risk of a severe confounding influence from genetic differences between populations across the sites. Another strength is the CVH construct, which provides a relatively holistic grouping of behavioural and biomedical risk factors for CVD. However, there were some limitations that warrant consideration. First, although the same methods were applied in all sites, the recruitment strategies had to be adapted to suit the local circumstances due to differences in registration systems. Ghanaian participants in Amsterdam, for example, were drawn from the Amsterdam Municipal Population register, whereas London participants were drawn mainly from Ghanaian organizations lists. It is possible that individuals who were not on the lists of these organizations differ in terms of socio-demographics, which might somewhat affect the representativeness of Ghanaian migrants in London and Berlin. Nevertheless, evidence suggests that most Ghanaians in Europe are affiliated with Ghanaian organizations [15]. We, therefore, anticipate that our study is representative of the Ghanaian population living in Berlin and London. Second, misclassification may have occurred in the PA and diet metrics, as both were measured based on self-reported questionnaires, which were not designed to evaluate an individual’s absolute amount of dietary intakes or PA [57]. Misclassification in the diet and PA metrics is likely to have overestimated the proportion of individuals in the ideal category due to social desirability bias. Moreover, the sodium intake (for the diet metric) was estimated from average sodium levels in dietary components reported, especially condiments, and did not include salt added while cooking or table salt. It has been shown previously that estimates of overall sodium intake estimated from a food frequency questionnaire (FFQ) do not correlate well with urinary sodium excretion. Thus, it is likely that there is a considerable underestimation of sodium levels within the diet metric. Sodium intake is, therefore, likely to have been underestimated. However, our approach on using approximations of the recommended consumption of fruits and vegetables, as well as the level of PA should not affect comparisons between sites. Third, smoking might have been under-reported in our study population due to a tendency towards responses considered socially desirable. The use of objective measures to confirm smoking status, such as urine analysis of nicotine metabolites, was unfortunately not conducted. In addition, the proportion of missing data in the diet metric may have biased our results. However, we used multiple imputation to improve the reliability of the results [21]. Finally, due to a lack of published data on CVH among the host population, in Amsterdam, Berlin, and London, we were unable to compare our results to the local population in the European sites. Despite these limitations, the RODAM study provides important data on living environment in determining CVH in the context of migration.

## Conclusion

Overall, the proportion of ideal CVH is extremely low in Ghanaians, especially among those living in urban Ghana and Ghanaian migrants in Europe. This study highlights the importance of living environment in determining CVH in the context of economic migration. Further studies need to be carried out to understand the causes, which may account for the differences in CVH across different living environments. This study shows a large gap between the prevalence of the ideal CVH and the AHA goal of improving cardiovascular health among Ghanaian migrants to Europe. When key determinants of poor ideal CVH have been identified, targeted CVH health promotion efforts at various levels (individual, social, political, and environmental) are required.

## Electronic supplementary material

Below is the link to the electronic supplementary material.

Supplementary material 1 (DOCX 320 kb)

## References

[1] World Health Organization Global status report on non-communicable diseases 2010 (2011), World Health Organization, Geneva. http://www.who.int/nmh/publications/ncd_report_full_en.pdf

[2] Go AS, Mozaffarian D, Roger VL, Benjamin EJ, Berry JD, Borden WB, Bravata DM, Dai S, Ford ES, Fox CS, Franco S, Fullerton HJ, Gillespie C, Hailpern SM, Heit JA, Howard VJ, Huffman MD, Kissela BM, Kittner SJ, Lackland DT, Lichtman JH, Lisabeth LD, Magid D, Marcus GM, Marelli A, Matchar DB, McGuire DK, Mohler ER, Moy CS, Mussolino ME, Nichol G, Paynter NP, Schreiner PJ, Sorlie PD, Stein J, Turan TN, Virani SS, Wong ND, Woo D (2013). Turner MB; American Heart Association Statistics Committee and Stroke Statistics Subcommittee. Heart disease and stroke statistics–2013 update: a report from the American Heart Association. Circulation.

[3] Nichols M, Townsend N, Scarborough P, Rayner M (2014). Cardiovascular disease in Europe 2014: epidemiological update. Eur Heart J.

[4] Gill PS, Kai J, Bhopal RS, Wild S, Stevens A, Raftery J, Mant J, Black and minority ethnic groups (2007). The epidemiologically based needs assessment reviews. Health care needs assessment.

[5] Global Burden of Disease Study 2013 Collaborators (2015). Global, regional, and national incidence, prevalence, and years lived with disability for 301 acute and chronic diseases and injuries in 188 countries, 1990–2013: a systematic analysis for the Global Burden of Disease Study 2013. Lancet.

[6] Celermajer DS, Chow CK, Marijon E, Anstey NM, Woo KS (2012). Cardiovascular disease in the developing world: prevalences, patterns, and the potential of early disease detection. J Am Coll Cardiol.

[7] Lloyd-Jones DM, Hong Y, Labarthe D, Mozaffarian D, Appel LJ, Van Horn L, Greenlund K, Daniels S, Nichol G, Tomaselli GF, Arnett DK, Fonarow GC, Ho PM, Lauer MS, Masoudi FA, Robertson RM, Roger V, Schwamm LH, Sorlie P, Yancy CW (2010). Rosamond WD; American Heart Association Strategic Planning Task Force and Statistics Committee. Defining and setting national goals for cardiovascular health promotion and disease reduction: the American Heart Association’s strategic Impact Goal through 2020 and beyond. Circulation.

[8] Folsom AR, Shah AM, Lutsey PL, Roetker NS, Alonso A, Avery CL, Miedema MD, Konety S, Chang PP, Solomon SD (2015). American Heart Association’s Life’s Simple 7: avoiding heart failure and preserving cardiac structure and function. Am J Med.

[9] Bambs C, Kip KE, Dinga A, Mulukutla SR, Aiyer AN, Reis SE (2011). Low prevalence of “ideal cardiovascular health” in a community-based population the heart strategies concentrating on risk evaluation (Heart SCORE) study. Circulation.

[10] Agyemang C, Addo J, Bhopal R, de Graft Aikins A, Stronks K (2009). Cardiovascular disease, diabetes and established risk factors among populations of sub-Saharan African descent in Europe: a literature review. Global Health.

[11] Adams G, Plaut VC (2005). The cultural grounding of personal relationship: friendship in North American and West African worlds. J Pers Soc Psychol.

[12] Dakubu MEK (1988). The languages of Ghana.

[13] Parra EJ, Marcini A, Akey J, Martinson J, Batzer MA, Cooper R, Shriver MD (1998). Estimating African American admixture proportions by use of population-specific alleles. Am J Hum Genet.

[14] Folsom AR, Yatsuya H, Nettleton JA, Lutsey PL, Cushman M, Rosamond WD, ARIC Study Investigators (2011). Community prevalence of ideal cardiovascular health, by the American Heart Association definition, and relationship with cardiovascular disease incidence. J Am Coll Cardiol.

[15] Agyemang C, Beune E, Meeks K, Owusu-Dabo E, Agyei-Baffour P, Aikins ADG, Amoah SK (2014). Rationale and cross-sectional study design of the research on obesity and type 2 diabetes among African Migrants: the RODAM study. BMJ Open.

[16] Agyemang C, Beune E, Meeks K, Addo J, Aikins ADG, Bahendeka S, Smeeth L (2017). Innovative ways of studying the effect of migration on obesity and diabetes beyond the common designs: lessons from the RODAM study. Ann N Y Acad Sci.

[17] Galbete C, Nicolaou M, Meeks KA, Aikins GDA, Addo J, Amoah SK, Smeeth L, Owusu-Dabo E, Klipstein-Grobusch K, Bahendeka S, Agyemang C, Mockenhaupt FP, Beune EJ, Stronks K, Schulze MB, Danquah I (2017). Food consumption, nutrient intake, and dietary patterns in Ghanaian migrants in Europe and their compatriots in Ghana. Food Nutr Res.

[18] Armstrong T, Bull F (2006). Development of the world health organization global physical activity questionnaire (GPAQ). J Public Health.

[19] Kaaks R, Riboli E (1997). Validation and calibration of dietary intake measurements in the EPIC project: methodological considerations. European Prospective Investigation into Cancer and Nutrition. Int J Epidemiol.

[20] Voedings Centrum NL. Groente en Fruit. Available from: http://www.voedingscentrum.nl/nl/gezond-eten-met-de-schijf-van-vijf/hoeveel-en-wat-kan-ik-per-dag-eten-/groente-en-fruit.aspx. Date accessed: 18/03/2017

[21] Rubin DB (2004). Multiple imputation for nonresponse in surveys.

[22] Obirikorang C, Osakunor DNM, Anto EO, Amponsah SO, Adarkwa OK (2015). Obesity and Cardio-metabolic risk factors in an urban and rural population in the Ashanti Region-Ghana: a comparative cross-sectional study. PLoS One.

[23] Agyemang C (2006). Rural and urban differences in blood pressure and hypertension in Ghana, West Africa. Public Health.

[24] Agyemang C, Nicolaou M, Boateng L, Dijkshoorn H, van de Born BJ, Stronks K (2013). Prevalence, awareness, treatment, and control of hypertension among Ghanaian population in Amsterdam, the Netherlands: the GHAIA study. Eur J Prev Cardiol.

[25] Misra A, Ganda OP (2007). Migration and its impact on adiposity and type 2 diabetes. Nutrition.

[26] Dolman RC, Wentzel-Viljoen E, Jerling JC, Feskens EJ, Kruger A, Pieters M (2014). The use of predefined diet quality scores in the context of CVD risk during urbanization in the South African Prospective Urban and Rural Epidemiological (PURE) study. Public Health Nutr.

[27] Kulshreshtha A, Goyal A, Dabhadkar K, Veledar E, Vaccarino V (2014). Urban-rural differences in coronary heart disease mortality in the united states: 1999–2009. Public Health Rep.

[28] Haas G, Parhofer K, Schwandt P (2010). Prevalence of cardiovascular disease risk factors in migrants participating in the PEP Family Heart Study, nuremberg. Int J Prev Med.

[29] Langellier BA, Garza JR, Glik D, Prelip ML, Brookmeyer R, Roberts CK, Peters A, Ortega AN (2012). Immigration disparities in cardiovascular disease risk factor awareness. J Immigr Minor Health.

[30] Jeemon P, Neogi S, Bhatnagar D, Cruickshank KJ, Prabhakaran D (2009). The impact of migration on cardiovascular disease and its risk factors among people of Indian origin. Curr Sci.

[31] Meeks KA, Stronks K, Adeyemo A, Addo J, Bahendeka S, Beune E, Owusu-Dabo E, Danquah I, Galbete C, Henneman P, Klipstein-Grobusch K, Mockenhaupt FP, Osei K, Schulze MB, Spranger J, Smeeth L, Agyemang C (2017). Peripheral insulin resistance rather than beta cell dysfunction accounts for geographical differences in impaired fasting blood glucose among sub-Saharan African individuals: findings from the RODAM study. Diabetologia.

[32] Brathwaite R, Addo J, Kunst AE, Agyemang C, Owusu-Dabo E, Aikins ADG, Beune E, Meeks K, Klipstein-Grobusch K, Bahendeka S, Mockenhaupt FP, Amoah S, Galbete C, Schulze MB, Danquah I, Smeeth L (2017). Smoking prevalence differs by location of residence among Ghanaians in Africa and Europe: the RODAM study. PLoS One.

[33] Agyemang C, Meeks K, Beune E, Owusu-Dabo E, Mockenhaupt FP, Addo J, de Graft Aikins A, Bahendeka S, Danquah I, Schulze MB, Spranger J, Burr T, Agyei-Baffour P, Amoah SK, Galbete C, Henneman P, Klipstein-Grobusch K, Nicolaou M, Adeyemo A, van Straalen J, Smeeth L, Stronks K (2016). Obesity and type 2 diabetes in sub-Saharan Africans—is the burden in today’s Africa similar to African migrants in Europe? The RODAM study. BMC Med.

[34] Beune EJ, Haafkens JA, Agyemang C, Bindels PJ (2010). Inhibitors and enablers of physical activity in multiethnic hypertensive patients: qualitative study. J Hum Hypertens.

[35] Walpole SC, Prieto-Merino D, Edwards P, Cleland J, Stevens G, Roberts I (2012). The weight of nations: an estimation of adult human biomass. BMC Public Health.

[36] Felton AM, Hall M (2015). Diabetes in Europe policy puzzle: the state we are in. Int Diabetes Nurs.

[37] Graciani A, León-Muñoz LM, Guallar-Castillón P, Rodríguez-Artalejo F, Banegas JR (2013). Cardiovascular health in a southern Mediterranean European country: a nationwide population-based study. Circ Cardiovasc Qual Outcomes.

[38] Wilsgaard T, Loehr LR, Mathiesen EB, Løchen ML, Bønaa KH, Njølstad I, Heiss G (2015). Cardiovascular health and the modifiable burden of incident myocardial infarction: the Tromsø Study. BMC Public Health.

[39] Chrichton GE, Elias MF, Davey A, Sauvageot N, Delagardelle C, Beissel J, Alkerwi A (2014). Cardiovascular health: a cross-national comparison between the Maine Syracuse Study (Central New York, USA) and ORISCAV-LUX (Luxembourg). BMC Public Health.

[40] Gaye B, Prugger C, Perier MC, Thomas F, Plichart M, Guibout C, Lemogne C, Pannier B, Boutouyrie P, Jouven X, Empana JP (2016). High level of depressive symptoms as a barrier to reach an ideal cardiovascular health. The Paris Prospective Study III. Sci Rep.

[41] Janković J, Marinković J, Stojisavljević D, Erić M, Vasiljević N, Janković S (2016). Sex inequalities in cardiovascular health: a cross-sectional study. Eur J Public Health.

[42] O’Flynn AM, McHugh SM, Madden JM, Harrington JM, Perry IJ, Kearney PM (2015). Applying the ideal cardiovascular health metrics to couples: a cross-sectional study in primary care. Clin Cardiol.

[43] Lachman S, Peters RJ, Lentjes MA, Mulligan AA, Luben RN, Wareham NJ, Boekholdt SM (2016). Ideal cardiovascular health and risk of cardiovascular events in the EPIC-Norfolk prospective population study. Eur J Prev Cardiol.

[44] Agyemang C, Kieft S, Snijder MB, Beune EJ, van den Born BJ, Brewster LM, Ujcic-Voortman JJ, Bindraban N, van Montfrans G, Peters RJ, Stronks K (2015). Hypertension control in a large multi-ethnic cohort in Amsterdam, The Netherlands: the HELIUS study. Int J Cardiol.

[45] Agyemang C, Bhopal R (2003). Is the blood pressure of people from African origin adults in the UK higher or lower than that in European origin white people? A review of cross-sectional data. J Hum Hypertens.

[46] Meeks KA, Freitas-Da-Silva D, Adeyemo A, Beune EJ, Modesti PA, Stronks K, Zafarmand MH, Agyemang C (2016). Disparities in type 2 diabetes prevalence among ethnic minority groups resident in Europe: a systematic review and meta-analysis. Intern Emerg Med.

[47] Doran T, Drever F, Whitehead M (2004). Is there a north-south divide in social class inequalities in health in Great Britain? Cross sectional study using data from the 2001 census. BMJ.

[48] Hilmers A, Hilmers DC, Dave J (2012). Neighborhood disparities in access to healthy foods and their effects on environmental justice. Am J Public Health.

[49] Walker RE, Keane CR, Burke JG (2010). Disparities and access to healthy food in the United States: a review of food deserts literature. Health Place.

[50] Agyemang C, Bhopal R, Bruijnzeels M (2005). Negro, Black, Black African, African Caribbean, African American or what? Labelling African origin populations in the health arena in the 21st century. J Epidemiol Community Health.

[51] Wardle J, Haase AM, Steptoe A, Nillapun M, Jonwutiwes K, Bellisie F (2004). Gender differences in food choice: the contribution of health beliefs and dieting. Ann Behav Med.

[52] Flegal KM, Carroll MD, Ogden CL, Curtin LR (2010). Prevalence and trends in obesity among US adults, 1999–2008. JAMA.

[53] Sandberg K, Ji H (2012). Sex differences in primary hypertension. Biol Sex Differ.

[54] van Genugten RE, Utzschneider KM, Tong J, Gerchman F, Zraika S, Udayasankar J, American Diabetes Association GENNID Study Group (2006). Effects of sex and hormone replacement therapy use on the prevalence of isolated impaired fasting glucose and isolated impaired glucose tolerance in subjects with a family history of type 2 diabetes. Diabetes.

[55] Guindon GE, Boisclair D. Past, current and future trends in tobacco use (2003) http://smtp.sesrtcic.org/tfo/files/articles-studies/6-past-current-and-future-treds-in-tobacco-use-who.pdf

[56] World Health Organization, and Research for International Tobacco Control (2008). WHO report on the global tobacco epidemic, 2008: the MPOWER package.

[57] Hebert JR, Clemow L, Obert L, Ockene IS, Ockene JK (1995). Social desirability bias in dietary self-report may compromise the validity of dietary intake measures. Int J Epidemiol.

