# Supplementary material for: Ideal cardiovascular health among Ghanaian populations in three European countries and rural and urban Ghana: the RODAM study
Source: Intern Emerg Med. 2018 Apr 17;13(6):845–56. doi: 10.1007/s11739-018-1846-6 (PMC6132772; doi:10.1007/s11739-018-1846-6)
Supplement: Supplementary file 1 — Supplementary material 1 (DOCX 320 kb) [file 11739_2018_1846_MOESM1_ESM.docx]

**Supplementary Table 1.** The AHA definitions of poor, intermediate, and ideal cardiovascular health for each metric^7^.

|  | **Cardiovascular health category** | | |
| --- | --- | --- | --- |
| **Metric** | **Poor** | **Intermediate** | **Ideal** |
| **Smoking** | Yes | Former ≤12 months | Never or quit >12 months |
| **Body mass index** | ≥30 kg/m^2^ | 25–29.9 kg/m^2^ | <25 kg/m^2^ |
| **Physical activity** | None | 1–149 min/week moderate intensity | ≥150 min/week moderate intensity |
|  |  | or 1–74 min/ week vigorous intensity | or ≥75 min/week vigorous intensity |
|  |  | or 1–149 min/week moderate + vigorous | or ≥150 min/week moderate + vigorous |
| **Diet** | 0–1 Components | 2–3 Components | 4–5 Components |
| **Total cholesterol** | ≥240 mg/dL | 200–239 mg/dL or treated to goal | <200 mg/dL |
| **Blood pressure** | SBP ≥140 or DBP ≥90 mm Hg | SBP 120–139 or DBP 80–89 mm Hg or treated to goal | <120/<80 mm Hg |
| **Fasting plasma glucose** | ≥ 126 mg/dL | 100–125 mg/dL or treated to goal | <100 mg/dL |
| **Overall CVH** | ≥ 1 metric in the poor category | ≥ 1 metric in the intermediate category and none in the poor category | all 7 CVH metrics in the ideal category |

BP refers to blood pressure, SBP refers to systolic blood pressure and DBP to diastolic blood pressure. Cholesterol refers to the level of total cholesterol in the blood, PA refers to physical activity, BMI refers to body mass index. The dietary components are: ≥ 450g of fruits and vegetables per day, ≥ Two 100g portions of fish a week, ≥ three 30 g equivalent servings of fibre-rich whole grains per day, ≤ 1500 mg sodium per day, ≤ 450 kcal sugar-sweetened beverages: (1 litre) per week. The five possible diet points that could be scored by an individual were defined as consuming: ≥ 450g of fruits and vegetables per day, ≥ Two 100g portions of fish a week, ≥ three 30 g equivalent servings of fibre-rich whole grains per day, ≤ 1500 mg sodium per day and 450 kcal sugar-sweetened beverages: (1 litre) per week.


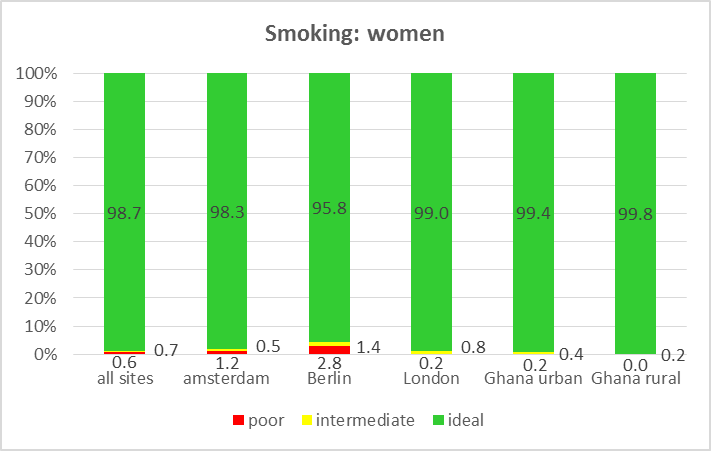

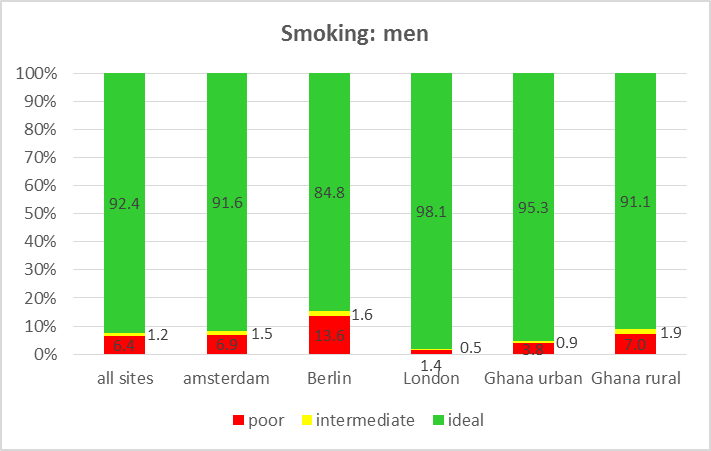


**B**

**A**

**Supplementary Fig. 1.** **Distribution of smoking categories (ideal, intermediate and poor) in women (A) and men (B).** Each bar represents one of the 5 study sites except the first bar, which is an average of all sites.


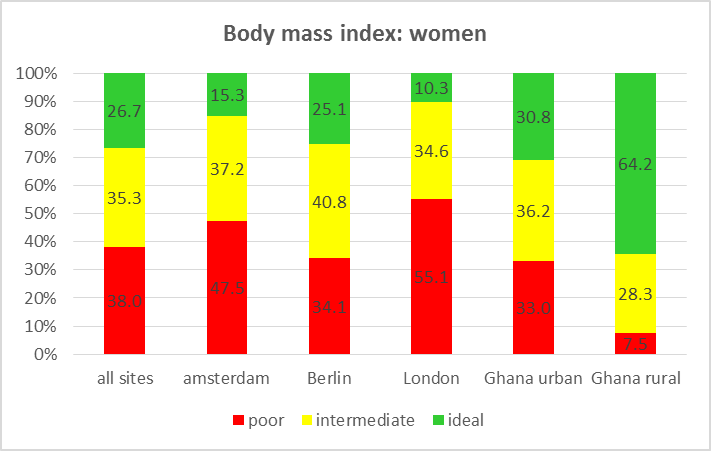

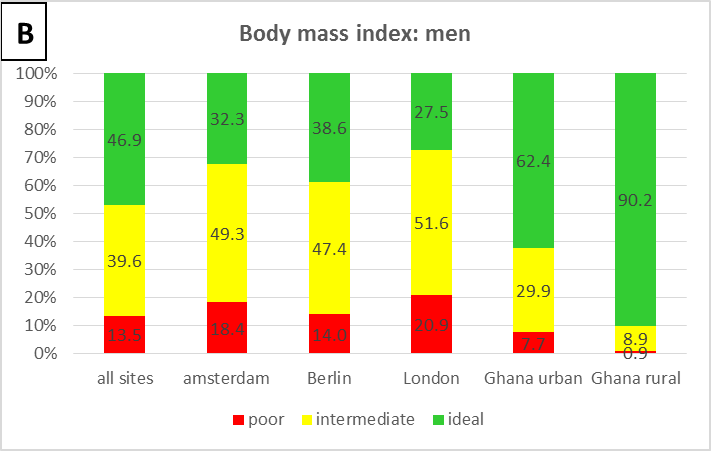


**A**

**Supplementary Fig. 2.** **Distribution of body mass index categories (ideal, intermediate and poor) in women (A) and men (B).** Each bar represents one of the 5 study sites except the first bar, which is an average of all sites.


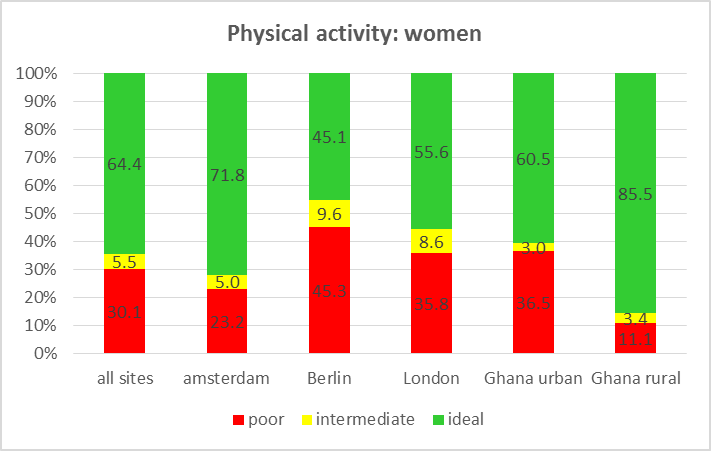

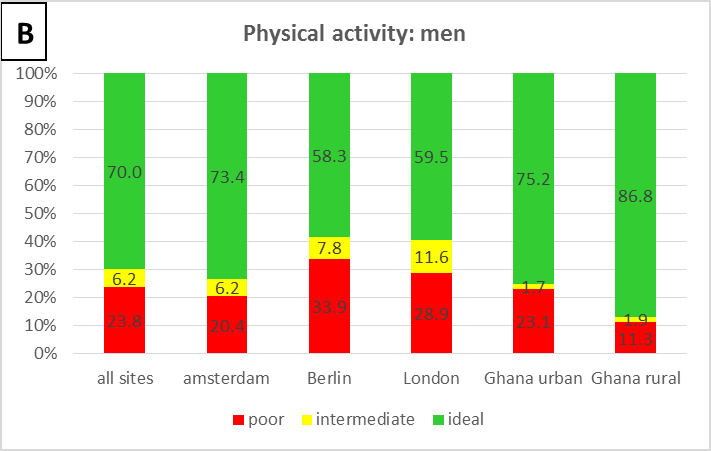


**A**

**Supplementary Fig. 3.** **Distribution of physical activity categories (ideal, intermediate and poor) in women (A) and men (B).** Each bar represents one of the 5 study sites except the first bar, which is an average of all sites.


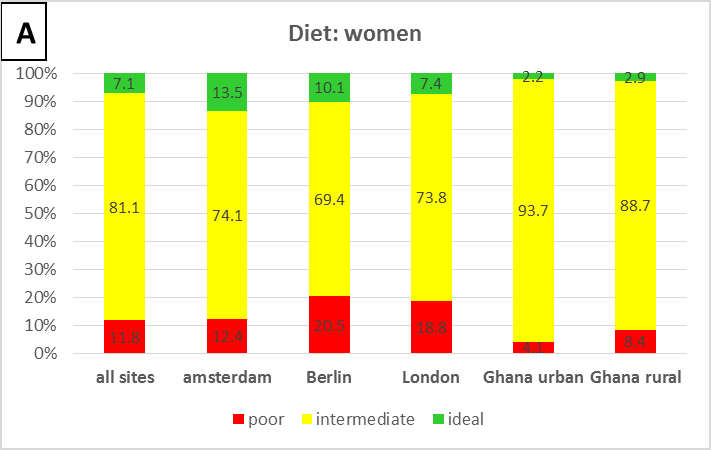

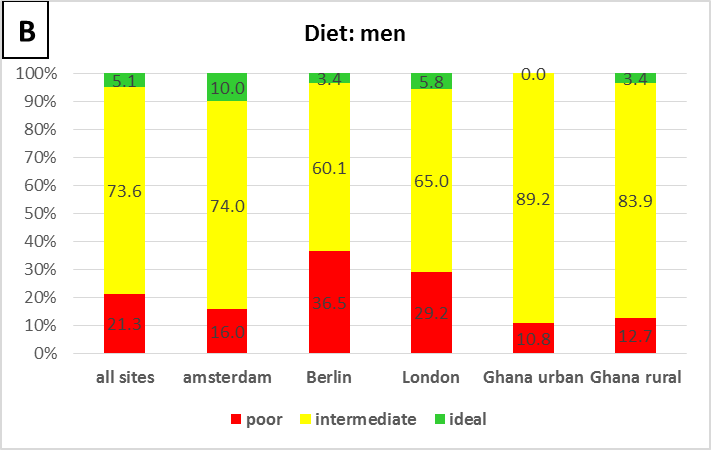


**Supplementary Fig. 4.** **Distribution of Diet (ideal, intermediate and poor) in women (A) and men (B).** Each bar represents one of the 5 study sites except the first bar, which is an average of all sites.


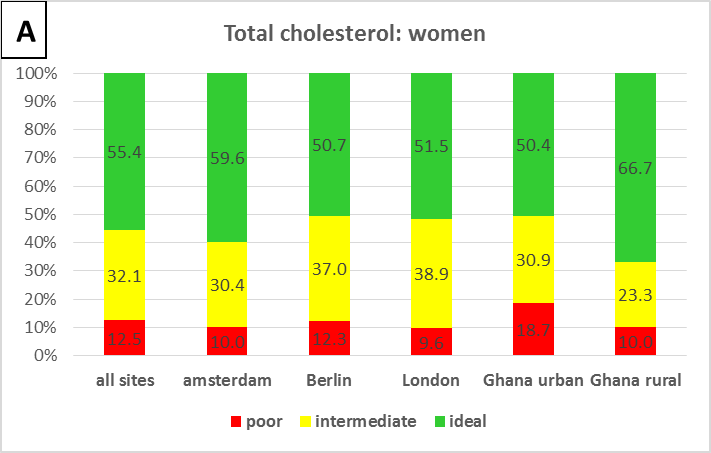

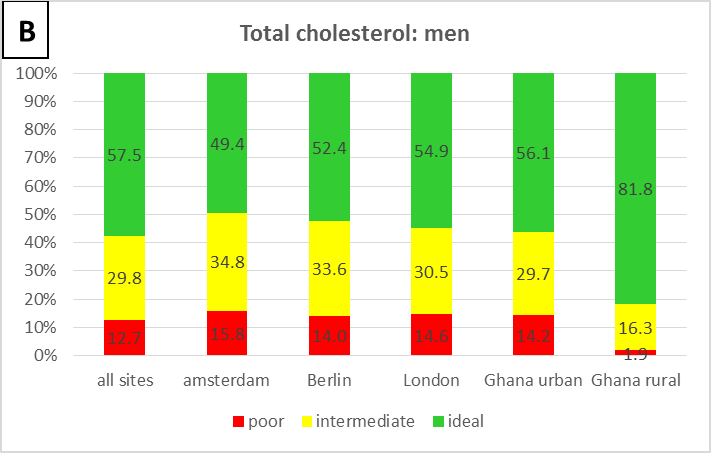


**Supplementary Fig. 5.** **Distribution of total cholesterol (ideal, intermediate and poor) in women (A) and men (B).** Each bar represents one of the 5 study sites except the first bar, which is an average of all sites.


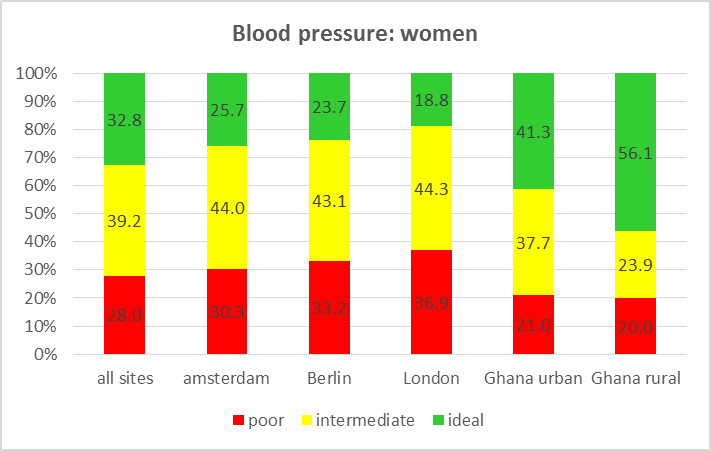

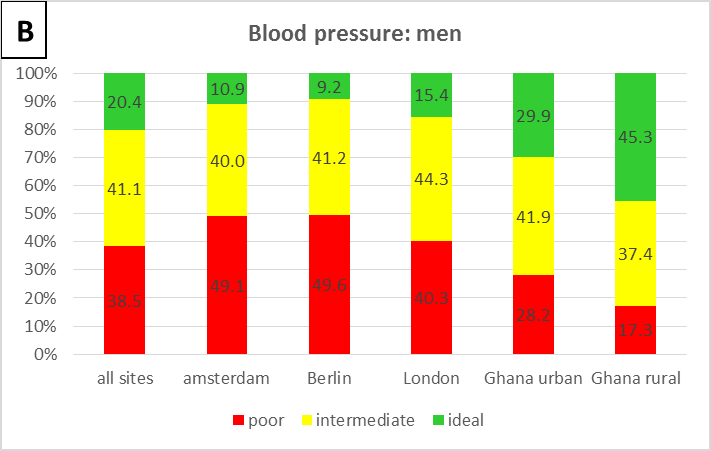


**A**

**Supplementary Fig. 6.** **Distribution of blood pressure (ideal, intermediate and poor) in women (A) and men (B).** Each bar represents one of the 5 study sites except the first bar, which is an average of all sites.


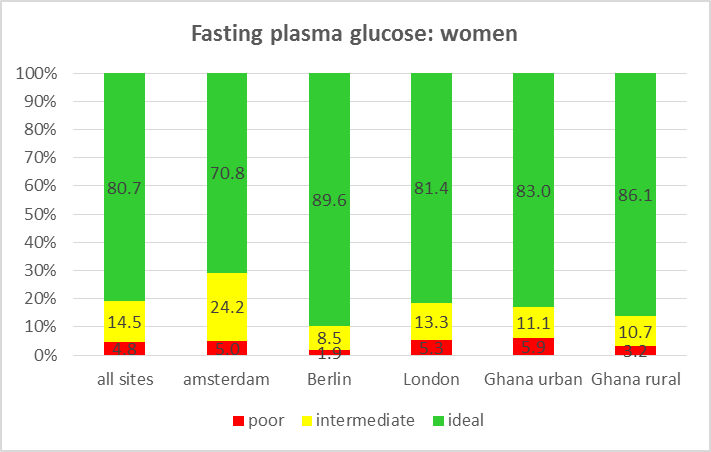

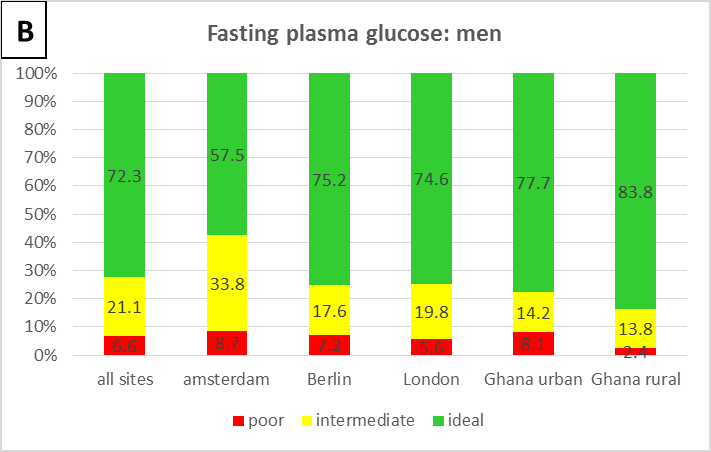


**A**

**Supplementary Fig. 7. Distribution of fasting plasma glucose (ideal, intermediate and poor) in women (A) and men (B).** Each bar represents one of the 5 study sites except the first bar, which is an average of all sites.
